# Chronic Myeloid Leukemia Presenting With T‐Lymphoid Blast Crisis in a Young Female With Marked Hyperleukocytosis: A Case Report

**DOI:** 10.1002/jha2.70213

**Published:** 2026-03-13

**Authors:** E. Somasundaram, R. P. Hasserjian, B. J. Aubrey, A. T. Fathi, H. Hock

**Affiliations:** ^1^ Department of Medicine Massachusetts General Hospital Boston Massachusetts USA; ^2^ Department of Pathology Massachusetts General Hospital Boston Massachusetts USA; ^3^ Department of Hematology and Medical Oncology Massachusetts General Hospital Boston Massachusetts USA

**Keywords:** blast crisis, CML, hyperleukocytosis, T cell

## Abstract

**Background:**

Chronic myeloid leukemia (CML) presenting in blast crisis (BC) is uncommon in the era of tyrosine kinase inhibitor (TKI) treatment, and T‐lymphoblastic BC is exceptionally rare. We report a case of T‐lymphoid BC presenting with extreme hyperleukocytosis.

**Case:**

A 37‐year‐old woman with previously diagnosed chronic phase CML who had declined therapy presented with dyspnea and constitutional symptoms. She was found to have diffuse lymphadenopathy and a markedly elevated white cell count (WCC) of 729 × 10^9^/L with 24% blasts. Bone marrow biopsy showed T‐lymphoblastic leukemia, *BCR::ABL1* fusion (p210), and a *RUNX1* gene mutation as well as an IKZF1 gene mutation. Staging revealed extensive nodal, splenic, renal, and ocular involvement. She underwent cytoreduction with hydroxyurea, prednisone, and dasatinib, followed by HyperCVAD induction. She achieved complete remission and proceeded to a sibling‐donor allogeneic stem cell transplant. A bone marrow biopsy 100 days after transplant showed complete remission without MRD assessed by PCR and flow cytometry. At 6 months post‐transplant, she remains disease‐free.

**Discussion:**

T‐lymphoid BC in CML is exceedingly rare, with limited knowledge of disease characteristics, molecular features, prognosis, and treatment outcomes. This case highlights the importance of early recognition, comprehensive molecular workup, and aggressive therapy. Despite historically poor outcomes, aggressive induction with BCR‐ABL1 kinase inhibition followed by transplant may offer durable disease control.

**Trial Registration:**

The authors have confirmed clinical trial registration is not needed for this submission.

## 1 Introduction

Chronic myeloid leukemia (CML) is a hematologic neoplasm characterized by clonal proliferation of myeloid cells that is driven by the t(9;22) chromosomal translocation, known as the Philadelphia (Ph) chromosome, which produces the constitutively active BCR‐ABL1 fusion tyrosine kinase [1]. CML is rare with an incidence of 1.0–1.5 per 100,000 and a median age of onset between 50 and 60 years [2]. While patients may present with symptoms, many are diagnosed incidentally on routine blood tests [3]. Approximately 85% of patients present in chronic phase (CP), characterized by elevated WCC displaying a full range of granulocytic differentiation along with the Ph chromosome [4]. Disease progression may follow a stepwise trajectory to accelerated phase (AP), including features such as circulating blasts, deranged cell counts, and secondary cytogenetic abnormalities [5], followed by blast crisis (BC) [6]. Of note, classification is evolving in that the current World Health Organization (WHO) classification, 5th edition, has eliminated the AP designation in favor of “high‐risk CP,” while the International Consensus Classification (ICC) has retained CML‐AP. Additionally, it is now recognized that the presence of any lymphoblasts in the blood or bone marrow (at least 5% per ICC) is indicative of lymphoid BC disease [7].

Before TKIs, the annual risk of progression to BC was up to ∼20% [8]. TKIs have dramatically reduced this to 1%–2% [9], and 8‐year survival now exceeds 85% [10]. BC now typically arises due to delayed diagnosis or TKI resistance and can present as either myeloid (∼70%) or lymphoid (∼30%)—with the latter almost always of B‐cell origin [11]. T‐lymphoid BC is exceedingly rare, and most knowledge has emerged from case reports [12, 13, 14]. Progression to BC has been linked to the accumulation of specific cytogenetic abnormalities, particularly trisomy 8, a second Ph chromosome, isochromosome 17q, and trisomy 19 [15].

Patients with CML in BC present similarly to those with acute leukemia, often with B symptoms, cytopenias, bone pain, or extramedullary involvement [16]. BC is associated with a poor prognosis. Some data suggest lymphoid BC is associated with a slightly better median survival compared to myeloid BC [17], although prognosis is overall dismal. Given its rarity in the TKI era, T‐lymphoid BC merits reporting. We describe a unique case of a 37‐year‐old woman who presented with T‐lymphoid BC featuring profound hyperleukocytosis (WCC 729 × 10^9^/L), the highest reported in this setting to date.

## 2 Case History

A 37‐year‐old woman with no significant medical history first presented to a local hospital with dyspnea and was found to have WCC 177 × 10^9^/L. She was noted to have had a prior WCC of 99 × 10^9^/L, although she had not undergone prior diagnostic evaluation. Repeat testing 3 months later showed WCC of 177 × 10^9^/L with 1% basophils, 2% blasts, 19% myelocytes, 4% promyelocytes, and an absolute neutrophil count (ANC) of 111 × 10^9^/L. Hemoglobin was 11.8g/dL, and platelets were 213 × 10^9^/L. Bone marrow biopsy showed a hypercellular marrow with increased myeloid‐to‐erythroid ratio and no increase in blasts. Fluorescence in situ hybridization (FISH) was positive for t(9;22)(q34;q11), and molecular studies showed a RUNX1 mutation (VAF 28.6%). Karyotype confirmed the Ph chromosome but showed no other cytogenetic abnormalities. She was diagnosed with CP CML but declined TKI therapy.

Two months later, she was found to have a left eye lesion concerning a choroidal neoplasm and was planning to see an oncology outpatient. However, she developed progressively worsening dyspnea, diffuse bony pain, and new cervical and axillary lymphadenopathy, at which point she presented to the MGH ED.

**FIGURE 1:**
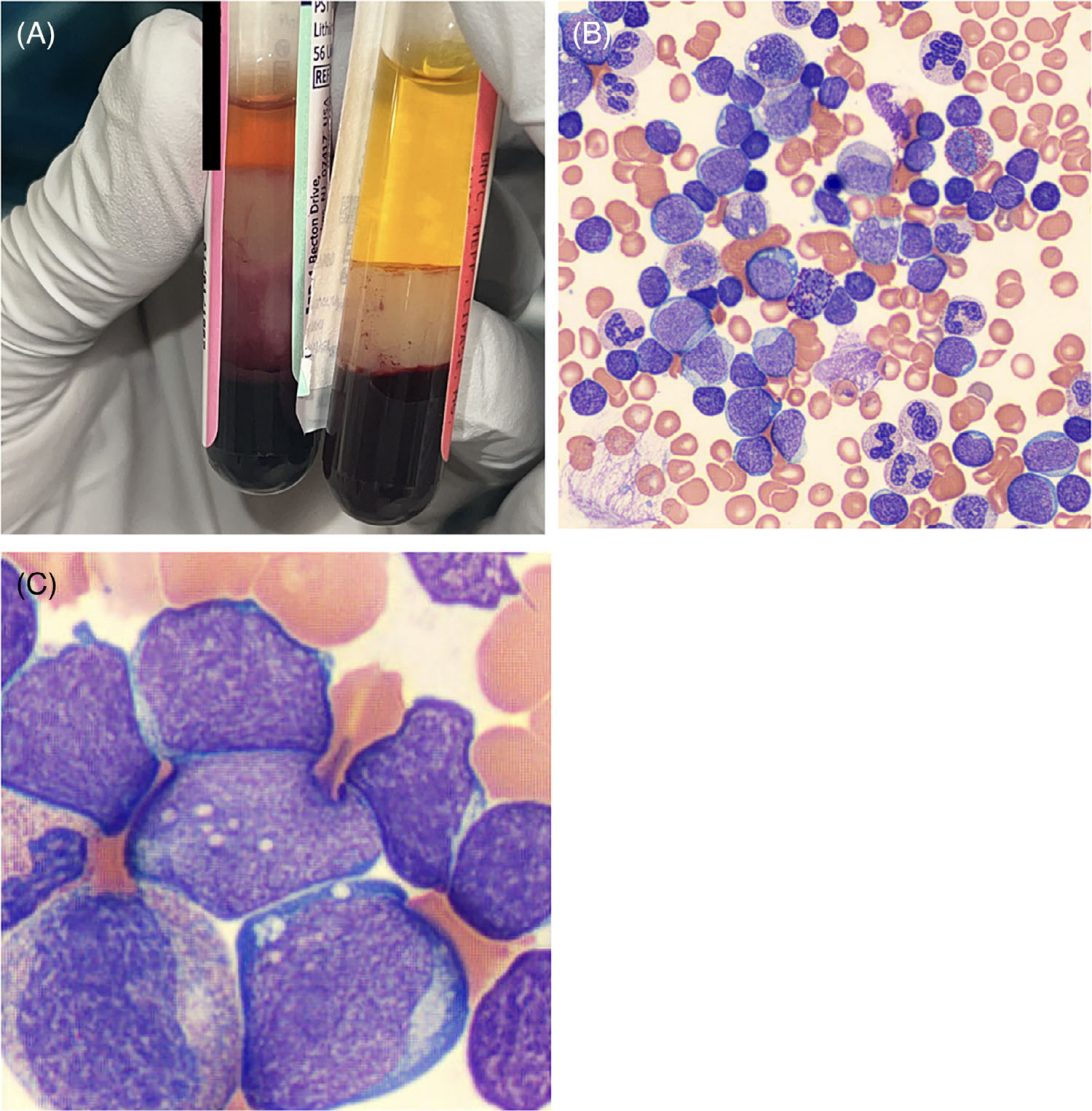
(A) Comparison of our patient's (left) spun‐down blood in a heparinized tube to a patient with a normal CBC (right). Gel layer is present in both. Buffy layer is notably expanded. (B) Overview of the peripheral smear. Notably, a high percentage of blasts is seen along with granulocytes in various stages of maturation. (C) Close‐up view of blasts.

Laboratory testing showed WCC 729 × 10^9^/L, hemoglobin 7.7g/dL, and platelets 103 × 10^9^/L. The differential showed 28% neutrophils, 38% lymphocytes, 7% immature granulocytes, and 24% blasts. Peripheral smear revealed increased blasts (Figure 1). Despite extreme leukocytosis, she only had subjective dyspnea and fatigue. Uric acid was elevated (10.9), but LDH, creatinine, electrolytes, and coagulation parameters were otherwise normal.

Bone marrow biopsy revealed > 90% cellularity with increased myeloid: erythroid ratio and abundant blasts, comprising 38% of the aspirate smear cells. Flow cytometry in the bone marrow showed partial CD34 and CD5 expression; full cCD3, CD7, and CD2 expression; and absent CD4 and CD8—consistent with T‐cell blasts. Myeloid and B‐cell lineage markers were negative (cMPO‐, CD117‐, CD11c‐, CD14‐, CD64‐, CD19‐ CD20‐ CD10‐, cCD79a‐). The blasts were also negative for myeloid markers (lysozyme, MPO, CD117, nonspecific esterase) and B‐cell markers (PAX5, CD19) by immunohistochemistry.

Quantitative reverse transcription PCR (qRT‐PCR) revealed the presence of *BCR::ABL1* (exons 13 and 2, respectively, p210 fusion) transcripts, with > 50% International Standard (IS) scale. Additional testing by NGS revealed a pathogenic RUNX1 mutation (p.S322Nfs*277, VAF 21.3%), an IKZF1 mutation (p.NV130KR, VAF 36.2%), and a WT1 variant (p.G19S, VAF 71.9%). Conventional karyotype showed the Ph chromosome as the sole cytogenetic abnormality. Staging CT/PET scan showed diffuse lymphadenopathy, splenomegaly, and FDG‐avid lesions in bilateral kidneys. CSF analysis was negative for malignant cells.

The patient was commenced on hydroxyurea, prednisone, and dasatinib 100 mg for initial cytoreduction. WCC decreased to < 15 × 10^9^/L within 1 week. She then began HyperCVAD 1A (cyclophosphamide, doxorubicin, vincristine, dexamethasone with intrathecal methotrexate/cytarabine) with dasatinib 140 mg. Post‐induction bone marrow biopsy showed complete morphologic remission and a normal karyotype. *BCR::ABL1* qRT‐PCR was 0.6% IS, but flow cytometry analysis showed no measurable residual T‐ALL. NGS panel showed no detectable RUNX1 and IKZF1 mutations, confirming that both were somatic mutations eradicated by treatment. In contrast, the WT1 mutations were undiminished (53% VAF), suggesting that it was a germline variant. Repeat ophthalmologic exam showed complete resolution of the choroidal lesion. Restaging PET‐CT showed resolution of LAD below the diaphragm and reduced FDG uptake in renal cortices. She then received HyperCVAD 1B consolidation (methylprednisolone, methotrexate, and cytarabine, along with intrathecal methotrexate and cytarabine) and continued dasatinib 140 mg.

The patient then underwent a matched related sibling donor peripheral blood stem cell transplant with fludarabine and ablative TBI conditioning. Dasatinib was held at the time of transplant and then resumed one month later following engraftment. Chimerism studies showed successful engraftment (Day 48: 100% CD33 and 93% CD3; Day 104: 100% CD33 and 98% CD3). At Day 100, a bone marrow biopsy showed no evidence of leukemia. Flow cytometry and *BCR::ABL1* qRT‐PCR were negative for residual disease. All prior mutations were absent. At 6 months from transplant, the patient remained in remission and good health.

## 3 Discussion

T‐lymphoid BC in CML is an extremely rare clinical entity. Fewer than 70 total cases have been reported in the literature [14]. In a single‐center review of 498 CML BC cases, only five (1%) were T‐cell phenotype [18]. Existing reports describe a wide age distribution (30–70 years) with possible male predominance [18, 19, 20, 21]. Extramedullary disease has been reported in 75% of previously reported cases of T‐lymphoid BC, usually in the CNS or lymph nodes [20]. Our patient had disease burden in the lymph nodes, spleen, and kidneys, along with presumed ophthalmic involvement.

A striking feature of this case was the hyperleukocytosis, with WCC peaking at 729 × 10^9^/L. Hyperleukocytosis is defined as WCC > 100 × 10^9^/L and is associated with leukostasis and TLS, though our patient did not have this. To our knowledge, this represents the highest WCC for any case of T‐lymphoid BC reported in the literature [22]. WCC levels exceeding 700 × 10^9^/L are exceedingly rare in adults and have been described primarily in pediatric/infant ALL, untreated CP CML, CLL, or T‐cell prolymphocytic leukemia [23, 24, 25, 26, 27].

Our patient had a typical T‐lymphoblastic immunophenotype, including cytoplasmic CD3+, CD2+, CD7+, partial CD34/CD5, TdT+, and CD4/CD8‐. Most T‐lymphoid BC cases, including ours, harbor the major *BCR::ABL1* breakpoint (p210) [14], rather than the minor (p190) breakpoint more commonly seen in de novo ALL [28].

Unlike most BC cases (∼70%), our patient had no additional cytogenetic abnormalities beyond t(9;22) [21]. In B‐lymphoid BC, 9p deletions are sometimes implicated, but no such associations have been described in T‐lymphoid BC [29]. Our patient also lacked a clonal T‐cell receptor gene rearrangement—a finding reported in other T‐lymphoid BC cases, distinguishing it from de novo T‐ALL [14]. This can also be seen in early T‐cell precursor (ETP) leukemia, but our patient's CD1a+ status would exclude this diagnosis.

Our patient had harbored a *RUNX1* mutation even in the CP of CML. RUNX1 is a hematopoietic stem cell differentiation transcription factor, and mutations of this gene have been associated with AML and T‐ALL [30] and previously reported in T‐lymphoid BC [31]. She later acquired a mutation in the transcription factor *IKZF1*, which is predicted to result in the exchange of two amino acids in zinc (Figure 1), involved in DNA binding. IKFZ1 mutations are common in B‐ALL and T‐ALL and have been linked to lymphoid BC and not typically seen in myeloid BC [32]. A broader understanding of the molecular basis of T‐lymphoid BC remains to be established. Table 1 compares our patient's molecular and cytogenetic information to prior cases.

**TABLE 1:** Comparison of our patient case across other reported T‐lymphoid BC cases reported in the literature. Extramedullary disease is typical in these presentations, often in lymph nodes. A complex karyotype is the norm, though our patient did not have this. Limited NGS is available, but the *RUNX1* pathogenic mutation present in our patient has been reported before.

**Authors**	**Cases**	**Karyotype**	**CD markers**	**NGS data**
This report	37F w/ EMD TC blast crisis EMD in lymph node, kidney, eye WCC: 729 × 10^9^/L, 24% blasts	46, XX t(9;22)(q34;q11) Bone marrow	CD34+, CD5+, cCD3+, CD7+, CD2+, CD4‐, CD8‐, cMPO‐, CD117‐, CD11c‐, CD14‐, CD64‐, CD19‐ CD20‐ CD10‐, cCD79a‐, lysozyme‐, nonspecific esterase‐, MPO‐, CD117‐, PAX5‐, CD19‐ IHC&flow, bone marrow	RUNX1 (36.3%), IKZF1 VUS (34.5%), NF1 splice variant (50.6%), and WT1 VUS (71.9%) Peripheral blood
Benlachgar et al. [33]	51M with EMD B&TC blast crisis EMD in lymph node WCC: 148 × 10^9^/L, 1% blasts	46, XY, t(9, 22)(q34.1;q11.2), (+6, +8, +10, +15, +19, +21 +19, +21)[6/30] Lymph node	CD34+, CD33+, MPO‐, CD10‐, CD79a+, CD19+, PAX5+, CD20‐, cycD1‐, MUM1‐, CD2+, CD3+, CD5+, CD7+, CD30‐, CD38+ Flow, lymph node	Not Reported
Shuyu et al. [31]	36M with ETP blast crisis WCC: 24.8 × 10^9^/L, 41%	48, XY, der(9)t(9;22)(q34;q11.2), +der(22)idic(22)(q11.2)t(9;22)x2, der(22) idic(22)(q11.2)t(9;22)[10]/49, idem, +der(22)idic(22)(q11.2)t(9;22)[10] Bone marrow	CD1a‐, CD2‐, cCD3+, sCD3‐, CD4+, CD5‐, CD7+, CD8‐, CD13+, CD33+, CD34+, CD117+, CD123+, HLA‐DR+, MPO‐, TdT‐ Flow, Bone marrow	TET2 Bone Marrow
Shuyu et al. [31]	57M with ETP blast crisis WCC: 60.4 × 10^9^/L, 5% blasts	46, XY, t(7;9;22)(p22;q34;q11.2)[17]/49, idem, +8, +19, +21[3] Bone marrow	CD1a‐, CD2+, cCD3+, sCD3‐, CD4‐, CD5dim, CD7+, CD8‐, CD13‐, CD33+, CD34‐, CD117+, CD123+, HLA‐DR‐, MPO‐, TdT‐ Flow, Bone marrow	None
Shuyu et al. [31]	84M with ETP blast crisis WCC: 325.5 × 10^9^/L, 88% blasts	46, XY, t(9;22)(q34;q11.2)[20] Bone marrow	CD1a‐, CD2‐, cCD3+, sCD3‐, CD4‐, CD5dim, CD7+, CD8‐, CD13+, CD33+, CD34+, CD117+, CD123+, HLA‐DR+, MPO‐, TdT+ Flow, Bone marrow	BCOR, RUNX1, STAG2 Bone Marrow
Matsuda et al. [34]	17M w/ EMD T&BC blast crisis EMD in CNS and neck soft tissue	47, XY, +6, t(9;22)(q34;q11)[3], 46, XY, t(9;22)(q34;q11)[8], 46, XY[9] Soft tissue mass	CD2+, CD3+, CD4+, CD5+, CD7+, CD8+, CD10+, CD13+, CD19‐, CD33‐, CD34‐, CD56+, HLA‐DR+ Flow, Soft tissue mass	Not reported
Zeng et al. [35]	44M w/ EMD TC blast crisis EMD in lymph node WCC: 170 × 10^9^/L	Not fully reported, BCR/ABL confirmed by FISH Lymph node	CD7+, CD3+, PAX5+, Bcl‐2+, TdT+, CD10‐, CD21‐ IHC, lymph node	Not reported
Raanani et al. [20]	45M w/ TC blast crisis WCC: 2.8 × 10^9^/L, 4% blasts	Not fully reported, FISH confirmed Ph1 chromosome Bone marrow	CD1 0%; CD2 60%; CD3 40%; CD4 2%; CD5 61%; CD7 66%; CD8 8%; TdT 60% Flow, Bone marrow	Not reported
Padhi et al. [36]	49M w/ TC EMD blast crisis EMD in lymph node WCC: 200 × 10^9^/L, 3% blasts	51‐53, XY, +8, +8, t(9;22)(34;q11.2), +15, +19, +21, +21, +der(22)t(9;22)[cp20] Lymph node	CD45+, cCD3+, sCD7+, CD43+, partial CD2+, CD13+, CD33+ TdTdim, CD1adim, sCD3‐, CD4‐, CD8‐, CD5‐, MPO‐, CD14‐, CD64‐, CD11b‐, CD19‐, CD20‐ Flow, lymph node	Not reported
Shah et al. [22]	33F w/ TC EMD blast crisis EMD in lymph node WCC: 561 × 10^9^/L, 2%–3% blasts	46, XX, t(9;22)(q34;q11.2) [4]/48, idem, +19, +der(22)t(9;22)[6] Lymph node	CD3+, CD5+, CD7+, CD8+, CD34+, CD45‐. CD4‐, CD16‐, CD56‐ Flow, Peripheral blood	ASXL1 pathologic, ATM VUS, RAD51D VUS Peripheral blood
Mongia et al. [19]	50M w/ myeloid and TC blast crisis WCC: 310 × 10^9^/L	46, XY, t(9; 22)(q34; q11) Bone marrow	CD34+, TdT+, cyCD3+, sCD3−, CD5+dim, CD1a‐, CD8‐ Flow, Bone marrow	WT1 (2 somatic mutations) Bone Marrow
Kim et al. [13]	72M w/ TC EMD blast crisis EMD in lymph node WCC: 427 × 10^9^/L, 0% blasts	46, XY, t(9;22)(q34;q11.2)[21]/59, XXYY, idem, add(1)(p32), +4, +6, +8, add(9)(p13), +10, +11, +14, +21, +22, [cp16]/46, XY[18] Bone marrow	CD1a+, CD2–, CD3–, CD4+, CD5+, CD7+, CD8–/+, CD10–, CD34+, TdT+, and BCL2+ IHC, lymph node	Not performed
Kim et al. [13]	32F w/TC EMD blast crisis EMD in lymph node WCC: 233 × 10^9^/L, 0% blasts	46, XX,?add(8)(p21), t(9;22) q11.2), der(9)del(9)(p13p22) (q34;q11.2)[1]/44, XX, idem, t(8;9;22)(p21;q34;q11.2), –6, –19[1]?; duplication der(22)(t;8;9;22)(p21;q34; of 9q34 Bone marrow	CD1a(bright)+, CD3(bright)+, CD4+, CD5+, CD8–, CD10–, CD34–, CD45(weak)+, and TdT+ IHC, lymph node	Not performed
Dorfman et al. [12]	72M w/ TC EMD blast crisis EMD in lymph nodes	Not fully reported, FISH confirmed Ph1 chromosome Lymph node	CD45+, CD34+, CD19‐, CD20‐, CD22‐, CD10‐, sIg‐, CD2‐, cCD3+, CD4‐, CD5‐, CD7+, CD8‐, CD43+, CD45RO+, TdT+, CD13+, MPO+, CD99+ IHC, lymph node	Not reported
Dorfman et al. [12]	35F w/ TC EMD blast crisis EMD in lymph nodes 18% blasts	Not fully reported, FISH confirmed Ph1 chromosome Lymph node	CD45+, CD34+, CD20‐, CD22‐, CD10+, sIg‐, cCD3+, sCD3‐, CD4‐, CD5+, CD7+, CD8‐, CD43+, CD45RO+, TdT+, CD13+, CD33+, MPO+, lysozyme+, CD99+ IHC, lymph node	Not reported
Dorfman et al. [12]	68M w/ TC blast crisis WCC: 217 × 10^9^/L, 90% blasts	Not fully reported, FISH confirmed Ph1 chromosome Bone marrow	CD45+, HLA‐DR‐, CD34‐, CD19‐, CD20‐, CD10+, CD2+, sCD3‐, CD5+, CD7+, CD45RO+, TdT+, CD11b‐, CD13‐, CD14‐, CD33‐, MPO‐, lysozyme‐, CD99+ Flow & IHC, Bone marrow	Not reported
Xia et al. [21]	28F w/ TC EMD blast crisis EMD in lymph nodes WCC: 40 × 10^9^/L	46, XX, t(9;22)(q34;q11)[20]/46, XX, t(10;11)(q11;p15)[20] Bone marrow	CD4+, CD7+, CD11b+, and CD38+, cCD3+, CD34+, and CD33+, CD19‐, CD11a‐, cCD79a‐, and cMPO‐ flow, bone marrow	Not reported
Al‐Janazreh et al. [37]	56M w/ TC EMD blast crisis EMD in lymph nodes	46, XY, t (9; 22) (q34; q11.2) [1]/49, XY, t (9; 22) (q34; q11.2), +19, +21, +der (22)t (9; 22) (q34; q11.2) [10]/46, XY [9] Bone marrow	CD5+; CD34+; CD33+; CD7+; cCD3dim Flow, BM	Not reported
Xu et al. [14]	24F w/ TC BC WCC: 188 × 10^9^/L, 10.5% blasts	46, XX, t(9;22)(q34;q11.2)[18]/46, idem, del(7)(p15)[2] Bone marrow	CD45+, cCD3+, CD1a+, CD2+, CD5+, CD7+, CD4+, CD8+, CD34+, and CD38+, CD19‐, cCD22‐, CD33‐, CD15‐, and cMPO‐ Flow, bone marrow	Not reported
Xu et al. [14]	66M w/ TC EMD blast crisis EMD in lymph nodes WCC: 57 × 10^9^/L	46, XY, del(5)(q13q33), t(9;22)(q34;q11.2), add(12)(p11.2), +14, −22[11]/51, idem, +8, +10, +11, +13, +19[9] Lymph node	CD45+, CD1a+, CD2+, sCD3−, cy‐CD3+, CD4−, CD5+, CD7+, CD8−, CD10−, CD19−, CD20−, CD38+, CD16−, CD57−, CD103−, CD13−, CD33+dim, CD34−, CD117+/−, and TdT+ Flow, lymph node	Not reported

T‐lymphoid BC carries a poor prognosis. In the pre‐TKI era, case series showed that nearly all patients died within 1 year despite aggressive chemotherapy, with a median survival of 4 months [20]. However, in patients with any type of BC, allogeneic stem cell transplant may improve survival: 5‐year survival was 43% in transplanted patients versus 13% in those who were not [38]. Accordingly, our patient underwent aggressive induction chemotherapy followed by bone marrow transplant.

Due to the rarity of this phenotype, long‐term relapse risk remains unclear. Factors favorable for our patient included complete cytogenetic remission post‐induction, lack of additional cytogenetic abnormalities, and successful donor engraftment. Historical data suggest that among BC patients achieving remission at transplant, the 3‐year relapse risk remains ∼46%, with most relapses occurring within the first year [39]. At 6 months post‐transplant, our patient remains in remission, feels in good health, and is cautiously optimistic about her prognosis for a historically aggressive disease.

## Author Contributions

E.S. was responsible for original draft preparation. All authors contributed equally toward investigation, methodology, and review and editing of this manuscript.

## Funding

The authors have nothing to report.

## Ethics Statement

An Institutional Review Board responsible for human subjects' research at Massachusetts General Hospital approved this research project.

## Consent

The patient was notified of this manuscript and provided informed consent to its publication.

## Conflicts of Interest

The authors declare no conflicts of interest.

## Supporting information




Supporting Information

## Data Availability

The data that support the findings of this study are available on request from the corresponding author. The data are not publicly available due to privacy or ethical restrictions.
