# Supplementary material for: Chronic Myeloid Leukemia Presenting With T‐Lymphoid Blast Crisis in a Young Female With Marked Hyperleukocytosis: A Case Report
Source: EJHaem. 2026 Mar 13;7(2):e70213. doi: 10.1002/jha2.70213 (PMC13483316; doi:10.1002/jha2.70213)
Supplement: Supplementary file 1 [file JHA2-7-e70213-s001.docx]

16. Wadhwa J, Szydlo RM, Apperley JF, Chase A, Bua M, Marin D, Olavarria E, Kanfer E, Goldman JM. Factors affecting duration of survival after onset of blastic transformation of chronic myeloid leukemia. *Blood.* 2002;99(7):2304-2309.

17.	Jain P, Kantarjian H, Jabbour E, Kanagal-Shamanna R, Patel K, Pierce S, Garcia-Manero G, Borthakur G, Ravandi F, O'Brien S, Cortes J. Clinical characteristics of Philadelphia positive T-cell lymphoid leukemias-(De novo and blast phase CML). *Am J Hematol.* 2017;92(1):E3-e4.

18.	Mongia A, Romano F, Ciullini Mannurita S, Peruzzi B, Bencini S, Parrini D, Fasano L, Fanelli A. A Rare Onset of T-Lymphoid Blast Crisis in Chronic Myeloid Leukemia with Two Distinct Blast Populations. *Hematol Rep.* 2024;16(3):413-420.

19.	Raanani P, Trakhtenbrot L, Rechavi G, Rosenthal E, Avigdor A, Brok-Simoni F, Leiba M, Amariglio N, Nagler A, Ben-Bassat I. Philadelphia-Chromosome-Positive T-Lymphoblastic Leukemia: Acute Leukemia or Chronic Myelogenous Leukemia Blastic Crisis. *Acta Haematologica.* 2005;113(3):181-189.

20.	Xia T, Yang Y, Li G, Chang J, Li J, Ren F, Ren W, Wang H, Xu Z. T-cell blast crisis of chronic myelogenous leukemia presented with coexisting p210 and p190 BCR-ABL transcripts and t(10;11)(q11;p15). *J Clin Lab Anal.* 2020;34(6):e23241.

21.	Shah S, Kundu R, Mishra R, Mukherjee S, Singh A. A rare case of Philadelphia-positive (P210BCR-ABL1) T-cell acute lymphoblastic leukemia/lymphoma associated with minimal residual disease persistence after intensive chemotherapeutic approaches. *Leukemia Research Reports.* 2024;21:100456.

22.	Chekol SS, Bhatnagar B, Gojo I, Hess JR. Leukopheresis for profound hyperleukocytosis. *Transfus Apher Sci.* 2012;46(1):29-31.

23.	Durzyński T, Konopka L, Traczyk Z. [Leukostasis syndrome in a case of chronic lymphocytic leukemia]. *Pol Merkur Lekarski.* 1999;6(31):30-31.

24.	Figueroa-Jiménez LA, Cabrera-Márquez AL, Báez-Díaz L, Cáceres-Perkins W. A patient with white blood cell counts more than a million: A diagnostic and therapeutic challenge. *Bisturi (S Juan).* 2016;2016:12-16.

25.	Jaime-Pérez JC, García-Arellano G, Herrera-Garza JL, Marfil-Rivera LJ, Gómez-Almaguer D. Revisiting the complete blood count and clinical findings at diagnosis of childhood acute lymphoblastic leukemia: 10-year experience at a single center. *Hematol Transfus Cell Ther.* 2019;41(1):57-61.

26.	Veigaard C, Aggerholm A, Hasle H, Kjeldsen E. Extreme hyperleukocytosis in a pediatric T-ALL patient with a rare translocation, t(7;19)(q35;p13), and submicroscopic deletions at 4q25, 7q33 and 10q23. *Leukemia Research Reports.* 2014;3(1):4-7.

27.	Kang ZJ, Liu YF, Xu LZ, Long ZJ, Huang D, Yang Y, Liu B, Feng JX, Pan YJ, Yan JS, Liu Q. The Philadelphia chromosome in leukemogenesis. *Chin J Cancer.* 2016;35:48.

28.	Ware AD, Wake L, Brown P, Webster JA, Smith BD, Duffield AS. B-Lymphoid Blast Phase of Chronic Myeloid Leukemia: A Case Report and Review of the Literature. *AJSP Rev Rep.* 2019;24(5):191-195.

29.	Sood R, Kamikubo Y, Liu P. Role of RUNX1 in hematological malignancies. *Blood.* 2017;129(15):2070-2082.

30.	E S, Xu J, Wang SA, Tang G, Jabbour EJ, Li S, You MJ, Medeiros LJ, Yin CC. Blast phase of chronic myeloid leukemia presenting as early T-cell precursor acute lymphoblastic leukemia. *Am J Clin Pathol.* 2025;163(2):231-239.

31.	Marke R, van Leeuwen FN, Scheijen B. The many faces of IKZF1 in B-cell precursor acute lymphoblastic leukemia. *Haematologica.* 2018;103(4):565-574.

32.	Jain P, Kantarjian HM, Ghorab A, Sasaki K, Jabbour EJ, Nogueras Gonzalez G, Kanagal-Shamanna R, Issa GC, Garcia-Manero G, Kc D, Dellasala S, Pierce S, Konopleva M, Wierda WG, Verstovsek S, Daver NG, Kadia TM, Borthakur G, O'Brien S, Estrov Z, Ravandi F, Cortes JE. Prognostic factors and survival outcomes in patients with chronic myeloid leukemia in blast phase in the tyrosine kinase inhibitor era: Cohort study of 477 patients. *Cancer.* 2017;123(22):4391-4402.

33.	Radujkovic A, Dietrich S, Blok H-J, Nagler A, Ayuk F, Finke J, Tischer J, Mayer J, Koc Y, Sorà F, Passweg J, Byrne JL, Jindra P, Veelken JH, Socié G, Maertens J, Schaap N, Stadler M, Itälä-Remes M, Tholouli E, Arat M, Rocha V, Ljungman P, Yakoub-Agha I, Kröger N, Chalandon Y. Allogeneic Stem Cell Transplantation for Blast Crisis Chronic Myeloid Leukemia in the Era of Tyrosine Kinase Inhibitors: A Retrospective Study by the EBMT Chronic Malignancies Working Party. *Biology of Blood and Marrow Transplantation.* 2019;25(10):2008-2016.

34.	Benlachgar N, Masrar A, Haidouri S, Harmouche H, Tazi Mezalek Z. Bi-lineage B- and T-lymphoid Extramedullary Blast Crisis at an Initial Presentation of Chronic Myeloid Leukemia: A Case Report and Literature Review of Extramedullary Blast Crisis. *Cureus.* 2023;15(11):e49348.

35.	Matsuda M, Morita Y, Shimada T, Miyatake J, Hirase C, Tanaka M, Tatsumi Y, Maeda Y, Kanamaru A. Extramedullary blast crisis derived from 2 different clones in the central nervous system and neck during complete cytogenetic remission of chronic myelogenous leukemia treated with imatinib mesylate. *Int J Hematol.* 2005;81(4):307-309.

36.	Zeng D-F, Chang C, Li J-P, Kong P-Y, Zhang X, Gao L. Extramedullary T-lymphoblastic blast crisis in chronic myelogenous leukemia: A case report of successful diagnosis and treatment. *Exp Ther Med.* 2015;9(3):850-852.

37.	Padhi P, Topalovski M, El Behery R, Cantu ES, Medavarapu R. A Rare Case of Chronic Myelogenous Leukemia Presenting as T-Cell Lymphoblastic Crisis. *Case Reports in Oncological Medicine.* 2018;2018(1):7276128.

38.	Al-Janazreh H, Abuzneid YS, Khamayseh I, Morabito F, Alqam B, Abusabbah RMF, Mustafa FK, Sarahneh S. An unusual case of T-cell acute lymphoblastic leukemia in a patient with BCR-ABL positive chronic myeloid leukemia and Gaucher disease. *Ann Med Surg (Lond).* 2021;68:102565.
